# Does Increasing Community and Liquor Licensees’ Awareness, Police Activity, and Feedback Reduce Alcohol-Related Violent Crime? A Benefit-Cost Analysis

**DOI:** 10.3390/ijerph10115490

**Published:** 2013-10-28

**Authors:** Héctor José Navarro, Anthony Shakeshaft, Christopher M. Doran, Dennis J. Petrie

**Affiliations:** 1 National Drug and Alcohol Research Centre (NDARC), University of New South Wales, Kensington, NSW 2052, Australia; E-Mail: h.navarro@unswalumni.com; 2 Hunter Medical Research Institute and the University of Newcastle, Room 3016, HMRI Building, Kookaburra Circuit, New Lambton Heights, NSW 2305, Australia; E-Mail: Christopher.Doran@hnehealth.nsw.gov.au; 3 Centre for Health Policy, Programs & Economics, Melbourne School of Population and Global Health, University of Melbourne, Level 4, 207 Bouverie Street, Carlton, VIC 3010, Australia; E-Mail: dennis.petrie@unimelb.edu.au

**Keywords:** alcohol-related violent crime, intervention, community, liquor licensees, police, feedback, benefit-cost analysis, economic

## Abstract

Approximately half of all alcohol-related crime is violent crime associated with heavy episodic drinking. Multi-component interventions are highly acceptable to communities and may be effective in reducing alcohol-related crime generally, but their impact on alcohol-related violent crime has not been examined. This study evaluated the impact and benefit-cost of a multi-component intervention (increasing community and liquor licensees’ awareness, police activity, and feedback) on crimes typically associated with alcohol-related violence. The intervention was tailored to weekends identified as historically problematic in 10 experimental communities in NSW, Australia, relative to 10 control ones. There was no effect on alcohol-related assaults and a small, but statistically significant and cost-beneficial, effect on alcohol-related sexual assaults: a 64% reduction in in the experimental relative to control communities, equivalent to five fewer alcohol-related sexual assaults, with a net social benefit estimated as AUD$3,938,218. The positive benefit-cost ratio was primarily a function of the value that communities placed on reducing alcohol-related harm: the intervention would need to be more than twice as effective for its economic benefits to be comparable to its costs. It is most likely that greater reductions in crimes associated with alcohol-related violence would be achieved by a combination of complementary legislative and community-based interventions.

## 1. Introduction

The negative consequences of alcohol misuse impose a significant burden of harm on society [1], primarily through increased alcohol-related social disruption, violence, crime and economic costs [2,3,4,5]. In Australia, alcohol-related crime alone accounted for 11% of the total social cost of alcohol misuse in 2004/2005, costing an estimated AUD$1.7 billion [5]. The largest single component of this cost (43%) was for policing [5]. Approximately half (40%–50%) of all alcohol-related crime is violent crime associated with heavy episodic drinking [6,7,8,9,10,11,12,13,14,15,16,17], the occurrence of which is concentrated on weekend nights and early mornings, and typically around late night licensed venues and areas with a high-density of licensed venues [8,12,14,18,19,20].

Multi-component interventions are highly acceptable to communities [21] and may be effective in reducing alcohol-related crime [14,22,23,24]. A recent 20 community cluster RCT, for example, called the Alcohol Action in Rural Communities (AARC) project, reported an estimated 40% reduction in alcohol-related verbal abuse in the 10 experimental, relative to 10 control, communities (*p* = 0.04) and an estimated 32% reduction in alcohol-related street offences (*p* = 0.06) [25]. The AARC project also showed that multi-component interventions can be cost-beneficial, estimating that for every AUD$1 invested in AARC, the value of benefits returned to experimental communities was between $1.37 and $1.75 [25].

Although the AARC study showed no statistically significant reduction in alcohol-related assaults or malicious damage, it evaluated the impact of 13 different interventions on a range of outcomes, rather than the impact of a multi-component intervention focused on a specific type of alcohol-related harm. Consequently, the aim of this study is to estimate the benefit-cost of a multi-component intervention for reducing alcohol-related violent crime, tailored to weekends identified as historically problematic in 10 experimental, relative to control, communities.

## 2. Method

### 2.1. Study Sample

This intervention trial was nested within the AARC project to utilise its cluster RCT design. Communities in New South Wales (NSW), Australia, were invited to participate if they had an urban-centre locality population between 5,000 and 20,000 (N = 27 communities) [26]; were at least 100 kilometres (km) away from a major urban centre, defined as a population of at least 100,000 (N = 24 communities); and were not known to be currently involved in any other large scale project aimed to assess or reduce alcohol-related harm (N = 20 communities). Communities of this population size were selected to ensure they were large enough to have sufficient police resources and a sufficiently high number of risky drinkers, to be able to detect any post-intervention changes as statistically significant.

### 2.2. Study Design

This study was a prospective, matched pairs RCT, with whole communities as the unit of randomisation and analyses. Given evidence of disproportionately high levels of alcohol-related harm among males [27], young people [27] and in Indigenous communities [28], the proportions of males, people aged 15–24 and Aboriginal and Torres Strait Islanders was obtained for each of the 20 communities, using the Australian Bureau of Statistics (ABS) 2001 Census of Population and Housing data [26]. Since the proportion of males and people aged 15–24 was similar across all communities, communities were listed, in decreasing order, according to the percentage of the population defined as Aboriginal or Torres Strait Islander and contiguous communities in the list were provisionally classified as matched pairs. Each pair was checked to ensure that they were at least 100 km apart, to minimise the risk of cross-contamination of any intervention effects between potential experimental and control communities. One community within each pair was then randomly allocated to the experimental group.

To account for different social, economic and geographic accessibility conditions, communities were categorised using the Socio-Economic Indexes for Areas (SEIFA) [29] and the ABS Accessibility/Remoteness Index of Australia (ARIA) [30]. SEIFA summarises the socioeconomic wellbeing of residents in a defined area, including average income, educational attainment, unemployment and indicators of wealth (e.g., owning a car, number of bedrooms in a dwelling). Low scores indicate high levels of socioeconomic disadvantage. ARIA scores incorporate the concept of remoteness based on the distance residents are required to travel by road to access services (e.g., goods, health care, social interaction). Low scores indicate greater accessibility (*i.e.*, less remote).

### 2.3. Data Sources

#### 2.3.1. Crime Data

NSW Police data on recorded criminal incidents in all 20 communities were obtained from the NSW Bureau of Crime Statistics and Research (BOCSAR) for the AARC study period of 1 January 2001 to 31 December 2009 [31]. Incidents were selected on the basis of the postcode in, and the date on, which they occurred. A criminal incident is defined as an activity detected by, or reported to, police, which: involves the same offender(s); involves the same victim(s); occurred at one location; occurred during one uninterrupted period of time; and falls into one offence category or incident type (e.g., “actual”, “attempted”, “conspiracy”) [32].

#### 2.3.2. Household Data

The number of households in the experimental communities was compiled from data provided by the respective local councils in 2008–2009.

### 2.4. Surrogate Measures

Routinely collected crime data facilitate comparisons between different periods over time in different communities, especially when reporting incidents on the basis of postcodes or Local Government Areas (LGAs) [31,33]. Using surrogate or proxy measures to identify crimes that are most likely to be alcohol-related [34,35,36,37,38], rather than relying on police recording practices which differ between communities and over time [33], minimises the impact of artefactual differences between communities in recording alcohol-involvement in crime [33,37]. Proxy measures of alcohol-related violent crime comprise types of criminal incidents that are typically alcohol-related and which occur at times that are highly likely to be associated with excessive alcohol consumption [31,35,37].

#### 2.4.1. Violent Crimes Typically Alcohol-Related

Alcohol-related violent crime incidents included in this study are: assaults (*common assault, actual bodily harm, grievous bodily harm (including malicious wounding), and assault to a police officer*); and sexual offences (*sexual assault, aggravated indecent assault, aggravated sexual assault, indecent assault*). Homicide offences, including murder and manslaughter, were excluded from this analysis because they occur too infrequently in these communities to provide any reliable estimate of the impact of the intervention.

#### 2.4.2. Times in Which Violent Crimes Are Most Likely to Be Alcohol-Related

Time of day and day of the week are useful in identifying violent crime incidents that are alcohol-related because these occur more frequently at night or early morning [18,31,39] and on weekends [8,12,14,18,19,20]. Indeed, night-time incidents of serious assaults have been used previously as a measure of alcohol-related crime [37,38]. This study uses time periods shown to be high-risk for alcohol-related crime in NSW: Friday 10 p.m.–Saturday 6 a.m., Saturday 6 p.m.–Sunday 6 a.m., and Sunday 10 p.m.–Monday 6 a.m. [31,36].

### 2.5. Selection of Problematic Weekends

For each of the 20 communities, the number of incidents of alcohol-related assaults was identified for all weekends from 2001 to 2007. Weekends that ranked in the top 30% for average number of alcohol-related assaults from 2001–2007, for both experimental and control communities, were identified as problematic (*i.e.*, an average of more than 3.8 incidents of alcohol-related assaults, from 2001 to 2007, for each weekend selected in a calendar year). Alcohol-related assaults were used to identify the problematic weekends because historically they have been the type of crime most frequently associated with alcohol-related violence in these communities [40].

### 2.6. Multi-Component Intervention

A co-ordinated effort between local councils, local media, alcohol licensees, liquor accords (a meeting of key stakeholders mandated by the NSW State Government) and the police in each experimental community was used to target the weekends identified as problematic in each community, commencing 23 May 2008 and concluding 31 December 2009. The intervention comprised four components. First, in the week leading up to the problematic weekend, the Mayor sent a letter to all alcohol licensees to make them aware that the impending weekend typically had high rates of violent crimes associated with alcohol and requesting that they be particularly vigilant in their responsible service of alcohol requirements, and that their security staff liaise with police in a timely manner to prevent any escalation of potential alcohol-related violence. For the first targeted weekend in each community, a “hot spot” map with the locations of alcohol-related crime incidents in the previous year was included with the letter.

Second, local media (print and/or radio) featured a story on the issue in the week leading up to the targeted weekend, based on a media release provided by the research team, to raise awareness in the community about the need for greater responsibility for those who would be drinking in licensed premises and in private homes on the coming weekend. Television media were not included in this strategy because their programs are regional and state-wide, rather than specific to each community, which would have increased the likelihood of contamination of intervention effects from the experimental to control communities.

Third, the local police agreed to increase their visibility by conducting foot or car patrols late at night and early in the morning on the Friday and Saturday of the problematic weekend, especially around licensed venues and the central business district. The extent of this increased police activity was determined solely by the Local Area Commander based on the number or police available and the need to attend to acute incidents in the district or neighbouring communities.

Fourth, in the week immediately after the problematic weekend, the number of incidents that occurred on that weekend, relative to the average number of incidents that had occurred on the same weekend between 2001 and 2007, was fed-back to the community (via local media) and key stakeholders (via the liquor accord meetings).

### 2.7. Statistical Analyses

All analyses were conducted using SAS version 9.2 [41]. Generalised estimating equations (GEEs), with a difference-in-difference base model, were estimated to evaluate the number of violent crimes associated with alcohol, separately for alcohol-related assaults and alcohol-related sexual offences, which occurred on the problematic weekends in the experimental, relative to the control, communities during the intervention period. To identify possible shifts in violent criminal incidents associated with alcohol from problematic to non-problematic weekends, as opposed to a net reduction, GEE models with the same specifications were estimated to evaluate the number of violent criminal incidents associated with alcohol which occurred on non-problematic weekends in the experimental, relative to the control, communities during the intervention period.

The GEE models were estimated as multivariate regressions to simultaneously control for the outcome data being clustered by communities (communities have observed and unobserved differences, such as population sizes and underlying rates of crime), autocorrelation in the data (since measures are longitudinal) and secular trends over time (such as seasonal effects). Negative binomial regressions were used, with counts of violent crimes associated with alcohol as the dependent variable, to avoid imposing the restrictive Poisson assumption that the mean and variance of the dependent variable are equal. For each GEE model, incidence rate ratios (IRR) are reported as an estimate of the relative difference in the percentage change in the number of violent crimes associated with alcohol in the experimental, compared to the control, communities.

### 2.8. Economic Costs and Benefits

The analysis of the economic benefits and costs of the intervention took a societal perspective. All values were inflated using the consumer price index to reflect average costs in 2008–2009 AUD [42].

#### 2.8.1. Intervention Costs

Intervention costs were estimated for: additional policing; media releases; letters to licensees; and the feedback to liquor accords. For additional policing, information was obtained by written follow-up surveys, distributed to police by the research team after each targeted weekend, on: police time (usual and overtime hours, including additional time spent on targeted weekend activities); activities other than routine weekend work; additional resources used in completing targeted weekend activities; and the rank of officers on duty on the targeted weekends. Year-specific average hourly wages and benefits for NSW police that corresponded to the relevant ranks of the officers on duty on each targeted weekend were used to cost the additional time spent by police on intervention activities and completing the post-weekend report. Costs for the media releases were obtained from local media outlets in the experimental communities, including: initial meetings inviting them to participate; drafting, printing and distribution of media releases; and broadcasting costs for a 30-second radio advertisement. Costs of the letters to licensees from the local Mayor’s office were obtained from the respective local councils, including the average time spent tailoring the standard letter and the required postage costs. The cost of written feedback to the liquor accord groups comprised the average time taken to draft the feedback and to communicate it by email after each targeted weekend.

#### 2.8.2. Intervention Benefits

The economic benefits were measured as the estimated total reductions per type of alcohol-related violent crime incidents, across all targeted weekends in all the experimental communities. These reductions were valued by combining estimates of the tangible and non-tangible benefits. Tangible benefits were obtained from existing estimates of the average cost per type of incident in NSW, comprising medical and lost output costs (AUD$7,500 per sexual offence and AUD$1,695 per assault in 2005 AUD) [43] and crime costs, such as law enforcement, court proceedings and correctional facilities (AUD$5,976 for sexual offences and AUD$1,695 for assaults in 2006 AUD) [44]. Non-tangible benefits comprised estimates of household willingness-to-pay (WTP), obtained from 3,017 surveys completed by randomly selected individuals (40% survey response rate) in the 20 AARC communities in 2005 [45,46]. Respondents identified that the maximum amount per annum their household would be willing to pay to reduce alcohol-related harm in their community was AUD$35.43 for the first 10% reduction and AUD$7.92 for the next 10% reduction [46].

#### 2.8.3. Benefit-Cost Ratios

A benefit-cost ratio, reflecting the comparison between intervention cost-savings to intervention costs, was estimated with the following equation:

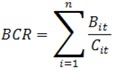
(1)
where *B_it_* is the benefit in communities *i* at time *t* and C*_it_* is the cost of the intervention in communities *i* at time *t*.

#### 2.8.4. Sensitivity and Uncertainty Analysis

One way sensitivity analyses were conducted to address the impact of variations in the effectiveness of the intervention. Uncertainty in benefit-cost outputs were evaluated by Monte Carlo simulation (2,000 iterations) using Ersatz version 1.13 [47], and 95% uncertainty intervals (UI) were calculated from the values resulting from the iterations [48].

## 3. Results

### 3.1. Community Characteristics and Number of Problematic Weekends

Table 1 delineates the comparable baseline characteristics of the experimental and control communities. A total of 115 problematic weekends were identified in the intervention period for the experimental communities and 116 for the control communities.

**Table 1:** Baseline characteristics of the experimental and control communities.

Covariate	Experimental (n = 10)	Control (n = 10)
	Mean (95% CI)	Mean (95% CI)
% young males (15–24 year olds)	6.0 (5.6–6.5)	5.9 (5.5–6.4)
% indigenous	5.7 (3.7–7.7)	5.4 (2–8.7)
Socioeconomic indicator (SEIFA disadvantage decile) [29]	3.5 (2.7–4.3)	3.3 (2.2–4.4)
Pubs/clubs ^a^	11.1 (8.3–14.0)	9.9 (7.1–12.6)
Other licensees ^a^	13.4 (9.9–17.0)	14.3 (10.3–18.4)
Police officers ^a^	14.2 (11.0–17.5)	22.4 (12.5–32.3)
Remoteness indicator (ARIA score)	2.9 (2.5–3.3)	2.9 (1.4–4.4)
% risky drinkers ^b^	26.0 (23.0–29.0)	29.1 (25.7–32.5)
Estimated number of risky drinkers ^c^	17,030	16,840
Total households in the 10 experimental communities	46,529	-

^a^ Rates per 10,000 population; ^b^ Proportion of respondents to the AARC baseline survey who reported a score of at least 8 on the Alcohol Use Disorders Identification Test (AUDIT), which represents the WHO category for hazardous and harmful drinking [25]; ^c^ Estimated number of risky drinkers (those who would score at least 8 on AUDIT).

### 3.2. Violent Crimes Associated with Alcohol before and after the Intervention

Table 2 summarises the impact of the intervention in the experimental, relative to the control, communities. For the problematic weekends targeted by the intervention, there was a statistically significant reduction in alcohol-related sexual offences (IRR 0.36; 95% CI: 0.14–0.96; *p* ≤ 0.05), equivalent to five fewer alcohol-related sexual offences (64% reduction) across all experimental communities. For the non-problematic weekends, there was a statistically significant reduction in alcohol-related assaults (IRR 0.81; 95% CI: 0.71–0.93; *p* ≤ 0.01), equivalent to 145 fewer assaults associated with alcohol across all experimental communities (19% reduction).

**Table 2:** Changes in the occurrence of violent crimes associated with alcohol in the experimental, relative to control, communities for problematic and non-problematic weekends during the intervention period (May 2008–December 2009).

Violent crime category	IRR	95% CI	*p*–value	Change	Change
			(≤)	(%)	(n)
***Problematic weekends targeted vs. not targeted***					
Assaults ^a^	1.00	0.66–1.53	0.96	1	0
Sexual offences	0.36	0.14–0.96	**0.05**	−64	5
***Problematic vs. non-problematic weekends***					
Assaults ^a^	0.81	0.71–0.93	**0.01**	−19	145
Sexual offences	0.77	0.47–1.27	0.31	−22	0

^a^ Alcohol-related homicide, murder, and manslaughter incidents were excluded because their low numbers were insufficient for analyses.

### 3.3. Benefit-Cost Ratios

Table 3 summarises the economic benefits and costs of targeting violent crimes associated with alcohol on problematic weekends. The additional average total cost of the intervention for the experimental communities for all targeted weekends was estimated as AUD$187,905. The value of the benefit of the intervention in achieving a statistically significant reduction in alcohol-related sexual offences in the experimental, relative to control, communities was estimated as AUD$4,126,123. The benefit-cost ratio was estimated as 22:1 and the net social benefit was estimated as AUD$3,938,218.

### 3.4. Sensitivity Analysis

Table 4 illustrates that if the intervention had reduced alcohol-related sexual assaults in the experimental communities by 10%, rather than the observed effect of 64%, then the estimated economic benefit would be $1,850,835.

**Table 3:** Economic benefits and costs of targeting violent crimes associated with alcohol on problematic weekends.

Intervention costs ^a^	Units	Total cost 2008–2009 (AUD)	
***Identifying targeted weekends***			
Time spent to identify problematic weekends	21 h	$958	
***Local Councils***			
Generating generic letter, identifying clubs/pubs and other licensees	4 h	$182	
Time spent to adapt template for each licensee each targeted weekend	5 min per licensee	$13,181	
Mail to each licensee each targeted weekend (stamp, printing and envelope)	AUD$0.74 per licensee	$3,348	
Mayor preparing and sending out letter for each licensee each targeted weekend	5 min per licensee	$5,565	
Time spent to generate “hot spot” map for initial targeted weekends	2 h per community	$847	
Printing “hot spot” map per licensee	AUD$0.30 per licensee	$118	
***Media***			
Generating generic media release	4 h	$182	
Tailoring media release pre-targeted weekend	20 min per targeted weekend	$1,749	
Printing & distribution of media releases for targeted weekends	1/3 page AUD$754	$82,186	
Radio media broadcasts for targeted weekends	AUD$423	$2,538	
***Police***			
Police visibility: extra vigilance, additional time patrolling, additional resources		$71,496	
Police time: filling out post-targeted weekend violent crime reports	1 h	$3,805	
***Liquor accords or community coalition groups***			
Generating and emailing targeted weekend reports	20 min per targeted weekend	$1,749	
**Total costs** **^b^**		**$187,905**	
**Benefits**	**Total cost 2008–2009 (AUD)**	**95% Uncertainty interval**	
***Targeted weekends***			
Alcohol-related sexual offences prevented (N = 5)	$74,005	($24,800–$82,800)	
***Households’ average willingness to pay ^a^***			
64% reduction in alcohol-related sexual offences	$4,052,118	($1,315,800–$4,660,800)	
**Total benefits** **^b^**	**$4,126,123**	($1,380,000–$4,720,000)	
Benefit-cost ratios	21.96:1		
Net social benefit	$3,938,218		

^a^ Source: Alcohol Action in Rural Communities project (AARC) [25]; Petrie *et al*. [46]; ^b^ Totals may not sum due to rounding.

**Table 4:** Sensitivity analyses for the statistically significant reduction in alcohol-related sexual offences in the experimental, relative to control, communities (2008/2009 AUD prices).

Outcome	Change (%) ^a^	Incidents prevented (n)	Average costs per incident ^b^	Average expenditure per incident ^c^	Average cost per incident	Average subtotal	Mean expected WTP per initial 10% reduction per household ^d^	Mean expected WTP per next 10% reduction per household^d^	Households (n)	Average total WTP	Average total
***Sexual offences***											
Observed	−64%	5	$8,354	$6,447	$14,801	$74,005	$39.46	$8.82	46,529	$4,052,118	$4,126,123
Decrease to	−25%	2	$8,354	$6,447	$14,801	$29,602	$39.46	$8.82	46,529	$2,451,613	$2,481,215
Decrease to	−10%	1	$8,354	$6,447	$14,801	$14,801	$39.46	-	46,529	$1,836,034	$1,850,835

^a^ Change in alcohol-related sexual offences in the experimental, relative to control, communities; ^b^ Source: Rollings [43], standardised to 2008/2009 AUD prices; ^c^ Source: Byrnes *et al*. [44], standardised to 2008/2009 AUD prices; ^d^ Source: Alcohol Action in Rural Communities project (AARC) [25]; Petrie *et al*. [46].

## 4. Discussion

### 4.1. Findings

Increasing community and liquor licensees’ awareness about violent crimes associated with alcohol, increasing police activity at high-risk times, and providing feedback on efforts to reduce those crimes appears to have no effect on alcohol-related assaults on problematic weekends, and a small, but statistically significant, effect on alcohol-related sexual assaults: a 64% reduction in the experimental communities which is equivalent to five fewer alcohol-related sexual offences. This reduction was not offset by an increase in alcohol-related sexual offences on non-problematic weekends. The economic analysis showed that the apparent reduction in alcohol-related sexual assaults was cost beneficial, achieving a net social benefit AUD $3,938,218, although this benefit-cost was only positive because it included the value communities place on reducing alcohol-related harm, rather than because the number of incidents averted outweighed the cost of the intervention. A statistically significant reduction in alcohol-related assaults on non-problematic weekends was also observed, which could be a diffusion effect (benefits of the intervention displaced from problematic to non-problematic weekends) or a consequence of the broader AARC project within which this trial was nested.

### 4.2. Methodological Considerations

A surrogate measure was used to quantify the relationship between alcohol and violent crime, rather than relying on incidents identified as alcohol-related by police. Although that means it is an indirect measure of alcohol involvement in the included crimes, surrogate measures to identify violent crimes associated with alcohol have been previously developed and applied in NSW and Australia [37], and the reliability of the specific surrogate measures used in this study have been found to be adequate [31,38].

That this trial was nested within the broader AARC trial [25] is both a strength and a potential limitation. This trial utilised the methodological strength of the cluster RCT design of AARC, which is the optimal evaluation design for being able to confidently conclude that a real change in the outcome occurred, that it was a consequence of the intervention and that the extent of change was statistically significant [49]. This nested design also makes it harder to ascribe the observed effects to this specific intervention rather than the broader AARC interventions. The data in Table 2, however, indicate that there was no effect on alcohol-related sexual assaults on weekends other than those targeted by this intervention, while the observed reduction in assaults on non-problematic weekends is consistent with the 13% reduction in alcohol-related assaults reported in the broader AARC project [25]. These findings support the validity of the outcome measures used and suggest that the observed outcomes are highly specific to this intervention.

Given it was not possible to blind the communities to their experimental or control status, it is also possible that the knowledge of being involved in an experimental community altered the reporting practices of police. It is unlikely, however, that police would significantly under-report the occurrence of violent crime, as they may do for less serious incidents, such as public disturbance. Although victims are known to under-report sexual assaults, especially in smaller communities where anonymity is a particular issue [50], the RCT design means it is most likely that rates of under-reporting were comparable in the experimental and control communities.

Although the economic analysis in this study used Australian data [43,44,46], it almost certainly under-estimates the true cost of alcohol-related sexual assaults because data are not readily available for a wide-range of costs, including: psychological and emotional suffering of the victim, the victim’s time; lost productivity of offenders; sentences other than imprisonment; suffering caused by false accusations; societal expenditure on increased private security; and the time for witnesses to attend a trial [51,52]. The extent of this under-estimation is likely to be substantial: based on the methods used in this analysis, the estimated cost per sexual assault in 2010 prices is AUD$8,769, compared to AUD$188,597 for a more comprehensive estimate from the United States [53], even though some of this difference would be explained by the different criminal justice systems.

The WTP estimates were generic to alcohol-related harm, as opposed to the specific alcohol-related crime categories used in this study. WTP estimates from the United States suggest that there is variation by crime type: USD$121 for serious assault; and USD$126 for rape and sexual assaults [54]. The most likely consequence of this limitation is a further under-estimation of the true extent of the value of the benefits of this intervention to communities, given some evidence that households are willing to pay more to reduce alcohol-related sexual offences than other types of alcohol-related harms [54].

### 4.3. Implications

This study is the first to estimate the benefit-cost of a multi-component intervention, specifically aimed at reducing rates of violent crime associated with alcohol. Recent evidence that types and rates of alcohol-related crime differ significantly across communities in Australia [31,55,56] provides a clear rationale for community involvement in recognising and responding to the specific nature of their own alcohol-related crimes [57,58,59], an approach that has also been shown to be highly acceptable to communities [21]. This study presents one method of engaging with communities to design, implement and evaluate a multi-component intervention, tailored to those weekends which have historically been most problematic for each of them in terms of violent crimes associated with alcohol.

The outcomes showed that increasing community and liquor licensees’ awareness about violent crime associated with alcohol, increasing police activity at high-risk times, and providing feedback on efforts to reduce those crimes appears to have no effect on alcohol-related assaults on problematic weekends and a statistically significant and cost-beneficial effect on alcohol-related sexual assaults. Nevertheless, the net number of alcohol-related sexual assaults averted was small (N = 5) and, as a consequence, the benefit-cost analysis was positive only because of the value communities place on reducing these harms (quantified by WTP): the intervention would have to be more than twice as effective for its economic benefits ($74,005) to be comparable to the cost of designing and implementing it ($187,905). The statistically significant reduction in assaults associated with alcohol on non-problematic weekends (equivalent to 145 fewer assaults) could be a diffusion effect, whereby the benefits of an intervention are dispersed from problematic to non-problematic weekends [60,61]. In this case, the net social benefit of the intervention would almost double to AUD $7,040,280, and the economic value of the benefits achieved ($896,680) would outweigh the cost of designing and implementing the intervention. The alternative explanation is that the reduction in assaults on non-problematic weekends was a consequence of the broader AARC project within which this trial was nested: AARC showed a non-statistically significant 13% reduction in alcohol-related assaults [25]. An independent replication study would clarify this uncertainty.

Assuming no diffusion effect, the modest outcome from this trial suggests either that the intervention was not optimally implemented or that the potential for community-based action to reduce alcohol-related violence, independently of other possible interventions, is limited. The latter explanation is more plausible. Although police did not always have the resources to fully implement increased visibility on every weekend, the other three components (mayoral letter to licensees, media awareness and post-weekend feedback) were successfully implemented on 115 problematic weekends in 10 communities over 19 months. In contrast, an Australian evaluation of restricting alcohol availability at high-risk times by forcing hotels to close at 3.30 a.m. instead of 5.00 a.m. in an urban city centre achieved a 37% reduction in assaults [39]. Although it is unclear if this 37% reduction would be achieved if earlier closing of hotels was implemented nationally, primarily because this was a retrospective evaluation conducted in one locality, it does highlight that legislative approaches may achieve comparable or improved intervention effects and economic outcomes, compared to community action.

## 5. Conclusions

It is most likely that optimal reductions in rates of violent crime associated with alcohol would be achieved by a combination of legislative-based policies and community-based action, which would recognise evidence that prevention-focused legislative approaches are highly cost-effective [62] and evidence that the types and rates of alcohol-related crime differ significantly between communities, even within a common over-arching legislative framework [31,55,56]. This study suggests that community-action approaches, in isolation from complementary legislative approaches, will have a modest impact on rates of violent crimes associated with alcohol, both in terms of reduced incidence and economic benefits.
